# Differential contributions of serotonergic and dopaminergic functional connectivity to the phenomenology of LSD

**DOI:** 10.1007/s00213-022-06117-5

**Published:** 2022-03-24

**Authors:** Timothy Lawn, Ottavia Dipasquale, Alexandros Vamvakas, Ioannis Tsougos, Mitul A. Mehta, Matthew A. Howard

**Affiliations:** 1 Department of Neuroimaging, Institute of Psychiatry, Psychology and Neuroscience, King’s College London, London, UK; 2 Medical Physics Department, School of Medicine, University of Thessaly, Larissa, Greece

**Keywords:** LSD, fMRI, REACT, Pharmacology, Receptor, Serotonin, Dopamine, Hallucination, Selfhood, Ego dissolution

## Abstract

**Rationale:**

LSD is the prototypical psychedelic. Despite a clear central role of the 5HT_2a_ receptor in its mechanism of action, the contributions of additional receptors for which it shows affinity and agonist activity remain unclear.

**Objectives:**

We employed receptor-enriched analysis of functional connectivity by targets (REACT) to explore differences in functional connectivity (FC) associated with the distributions of the primary targets of LSD—the 5HT_1a_, 5HT_1b_, 5HT_2a_, D1 and D2 receptors.

**Methods:**

We performed secondary analyses of an openly available dataset (*N* = 15) to estimate the LSD-induced alterations in receptor-enriched FC maps associated with these systems. Principal component analysis (PCA) was employed as a dimension reduction strategy for subjective experiences associated with LSD captured by the Altered States of Consciousness (ASC) questionnaire. Correlations between these principal components as well as VAS ratings of subjective effects with receptor-enriched FC were explored.

**Results:**

Compared to placebo, LSD produced differences in FC when the analysis was enriched with each of the primary serotonergic and dopaminergic receptors. Altered receptor-enriched FC showed relationships with the subjective effects of LSD on conscious experience, with serotonergic and dopaminergic systems being predominantly associated with perceptual effects and perceived selfhood as well as cognition respectively. These relationships were dissociable, with different receptors showing the same relationships within, but not between, the serotonergic and dopaminergic systems.

**Conclusions:**

These exploratory findings provide new insights into the pharmacology of LSD and highlight the need for additional investigation of non-5HT_2a_-mediated mechanisms.

**Supplementary Information:**

The online version contains supplementary material available at 10.1007/s00213-022-06117-5.

## Introduction

Psychedelic compounds profoundly modulate conscious experience. Lysergic acid diethylamide (LSD) is the prototypical psychedelic and its serendipitous discovery in 1943 (Hofmann 1979) prompted significant research into the therapeutic efficacy of psychedelics for diverse disorders including depression (Pahnke et al. 1969, 1970), alcoholism (Krebs and Johansen 2012) and chronic pain (Kast and Analgesia 1964). However, legal restriction halted this line of inquiry in the early 1970s (Nutt 2015). Recently, there has been a psychedelic renaissance in neuroscience with a variety of these compounds being re-investigated with the benefit of modern techniques.

Neuroimaging approaches examining the neural correlates of the LSD experience have demonstrated numerous changes in the brain at rest. Specifically, increased cerebral blood flow in the primary visual cortex (Carhart-Harris et al. 2016), reduced functional network integrity (Tagliazucchi et al. 2016; Carhart-Harris et al. 2016), increased global connectivity (Tagliazucchi et al. 2016; Carhart-Harris et al. 2016; Preller et al. 2018), increased sensitivity of dynamic responses to perturbation (Jobst et al. 2021) and that altered dynamic integration and segregation are non-uniform in time (Luppi et al. 2021). Whilst these have revealed key insights into systems-level mechanisms, such approaches are subject to the inherent inability of pharmacological fMRI to provide insight into the molecular underpinnings of BOLD activity.

LSD shows high affinity and agonist activity at the 5-HT_1A/B_, 5-HT_6_, 5-HT_7_ as well as D1 and D2 receptors (Marona-Lewicka et al. 2002; Nichols 2004; Passie et al. 2008; de Gregorio et al. 2016a). Serotonin and dopamine provide widespread neuromodulatory control over the brain (Marder 2012; Avery and Krichmar 2017; Shine et al. 2019). Crucially, they can tweak the gain of receptive neuronal populations through altering their electrical and synaptic properties, thus also affecting subsequent downstream inter-regional communication (Aston-Jones and Cohen 2005). As such, these systems are well placed to mediate the profoundly altered network architecture of the brain under LSD, though the multiplicity of receptors makes clearly delineating their contributions challenging.

To date, LSD has been primarily studied with a focus on the 5HT_2a_ receptor and pharmacological manipulation has provided strong evidence for its primacy in the psychedelic state of consciousness. This is principally evidenced by the ability of ketanserin, a non-selective 5HT_2_ antagonist with the highest affinity for 5HT_2A_ receptors, to reduce both subjective reports and neural measures associated with LSD administration (Preller et al. 2017, 2018; Kraehenmann et al. 2017). Despite offering important insights into causality, these studies remain incapable of fully delineating the underlying mechanisms. Specifically, whilst it can be claimed that 5HT_2a_ agonism is necessary for the majority of the effects of LSD, it is clearly not sufficient; the overall functioning of the brain relies on a broad array of neurotransmitters across highly complex neural systems. By blocking the 5HT_2a_ receptor, any effects of other receptor systems that interact causally downstream of this are also blocked and thus cannot be investigated. 5HT_1a_ agonism and antagonism have been shown to diminish visual effects of psilocybin and bolster the effects of N,N-dimethyltryptamine respectively (Strassman 1995; Pokorny et al. 2016). Furthermore, a host of preclinical studies have implicated actions of LSD at the 5HT_1a_, 5HT_1b_, D1 and D2 receptors (Nichols 2016; de Gregorio et al. 2016a, 2021) in a complex pleiotropic manner (de Gregorio et al. 2016b). Whilst activation of other serotonergic and dopaminergic targets alone may not be sufficient to produce the primary facets of the psychedelic state, the actions of LSD on these receptors in the presence of 5HT_2a_ agonism likely provide a more complete insight into both how these circuits function as well as how LSD can modulate them.

Clear and definitive delineation of these mechanisms is a key goal if psychedelic-based pharmacotherapy is to be maximally exploited for therapeutic benefit, especially with regard to the potential development of novel compounds that more selectively interact with a subset of these receptors. To this end, we employed receptor-enriched analysis of functional connectivity by targets (REACT (Dipasquale et al. 2019)) to further delve into the neural correlates of LSD by incorporating molecular information about the neurotransmitter systems into an existing resting-state fMRI dataset (Carhart-Harris et al. 2016). Specifically, we mapped the pharmacodynamic effects of LSD onto the distribution of its primary targets: the 5HT_1a_, 5HT_1b_, 5HT_2a_, D1 and D2 receptors. We then characterised the differences in these whole-brain maps of FC informed by the distribution of each receptor, alongside their experiential correlates, under conditions of LSD and placebo.

## Methods

### Participants

In this work, we employed a subset (15 healthy controls (age: 30.5 ± 8.0 years, M/F: 4/11)) of a previously published dataset (Carhart-Harris et al. 2016), made publicly available on the OpenNeuro data repository (https://doi.org/10.18112/openneuro.ds003059.v1.0.0) (see Carhart-Harris et al. (2016) for full inclusion and exclusion criteria). The original study received approval from the National Research Ethics Service committee London-West London and was conducted in accordance with the revised declaration of Helsinki (2000).

### Study design, scene and dosing

Participants undertook two scanning days (LSD and placebo) separated by at least 2 weeks in a balanced-order within-subjects design. Participants received either LSD (75 μg in 10 mL saline) or placebo (10 mL saline) infused over a 2-min period through a cannula inserted into the antecubital fossa. This was followed by a period of acclimatisation inside a mock MRI scanner for approximately 60 min, intended to help attenuate the potentially anxiogenic effects of the real MRI scanning environment. Subjective drug effects were reported to take effect within 5 to 15 min and to peak between 60 to 90 min. A plateaued drug effect was generally maintained for approximately 4 h post-dosing. MRI scanning was undertaken approximately 70 min post-dosing.

### MRI acquisition

Imaging data was collected on a 3 T GE HDx system. Anatomical images were 3D fast spoiled gradient echo scans in an axial orientation, with field-of-view = 256 × 256 × 192 mm and matrix = 256 × 256 × 192 yielding 1-mm isotropic voxel resolution: TR/TE = 7.9/3.0 ms; inversion time = 450 ms; flip angle = 20°. Three BOLD-weighted eyes-closed fMRI resting-state runs were acquired using a gradient echo-planar imaging sequence, TR/TE = 2000/35 ms, field-of-view = 220 mm, 64 × 64 acquisition matrix, parallel acceleration factor = 2, 90° flip angle. Thirty-five oblique axial slices were acquired in an interleaved order, each 3.4 mm thick with no slice gap yielding 3.4-mm isotropic voxels. Each run lasted 7 min and, as the second run included listening to music, here we analyse only the first and third (Carhart-Harris et al. 2016).

### Subjective report of phenomenology

To characterise the phenomenology of the LSD experience subjects completed the 11-dimension Altered States of Consciousness (ASC) questionnaire at the end of the LSD dosing day (Studerus et al. 2010). The sub-scores measured within the ASC are the experience of unity, spiritual experience, blissful state, insightfulness, disembodiment, impaired control and cognition, anxiety, complex imagery, elemental imagery, audio-visual synaesthesia and changed the meaning of percepts. Visual analogue scale (VAS) ratings of subjective effects were also measured after each LSD run. These questions included (1) “With eyes closed, I saw patterns and colours”; (2) “With eyes closed, I saw complex visual imagery” and (3) “I experienced a dissolving of myself or ego”. VAS questions were rated between “no more than usual” and “much more than usual”.

### Principal components analysis of subjectively reported experiences

We employed principal components analysis (PCA) for dimensionality reduction to investigate whether components capturing multiple related facets of the subjective LSD experience could be derived and may show relationships with receptor-enriched FC. We chose to exclude anxiety given the lack of statistically significant difference between LSD and placebo conditions (Carhart-Harris et al. 2016). The suitability of the data to PCA was tested using Kaiser–Meyer–Olkin Measure of Sampling Adequacy and Bartlett’s test of sphericity, to respectively measure the proportion of variance within the variables that might be caused by underlying factors and test the hypothesis that the correlation matrix is an identity matrix. Principal components were retained under the eigenvalue-one criterion. Following the generation of the principal components, additional rotation of the factor axes was applied to determine a simplified and more interpretable pattern. Varimax rotation was employed as the rotation method to ensure orthogonality.

### Image pre-processing

The present work makes use of data that had already undergone pre-processing prior to uploading to the OpenNeuro repository. Briefly, these steps were (1) removal of the first three volumes; (2) de-spiking; (3) slice time correction; (4) motion correction; (5) brain extraction; (6) rigid body registration to anatomical scans; (7) non-linear registration to 2 mm^3^ MNI template; (8) scrubbing; (9) spatial smoothing (FWHM) at 6 mm; (10) band-pass filtering between 0.01 and 0.08 Hz; (11) linear and quadratic de-trending; (12) regressing out 9 nuisance regressors (6 motion-related (3 translations and 3 rotations)) and 3 anatomically related (ventricles, draining veins and local white matter)) (for full details see Carhart-Harris et al. (2016).

### Population-based molecular templates

**Fig. 1:**
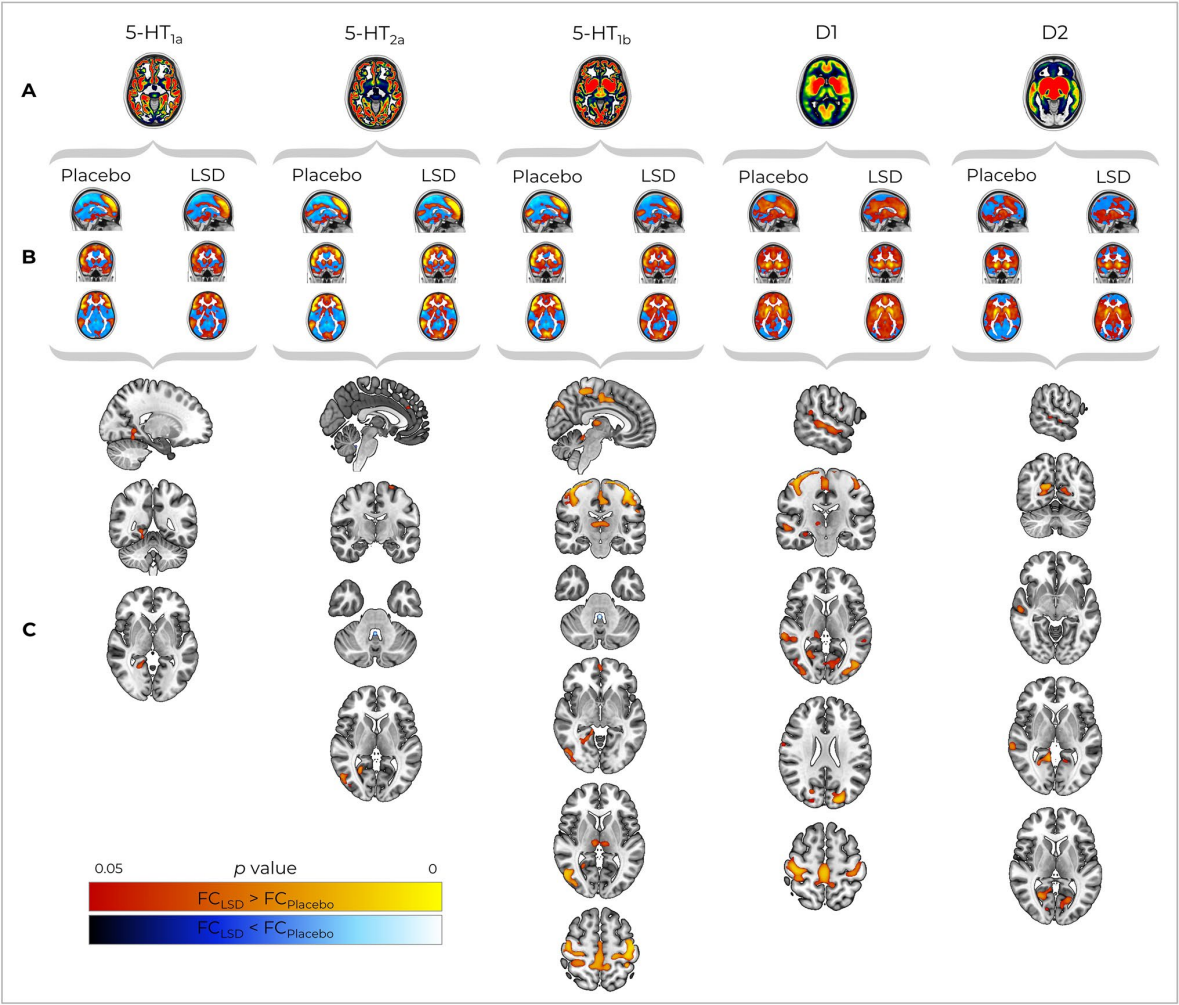
**A** Maps of the different neurotransmitter receptors employed within the REACT analysis; **B** receptor-enriched FC maps averaged across subjects for the LSD and placebo conditions; **C** the results of paired *t*-tests comparing FC between LSD and placebo conditions within the different receptor-enriched maps. All images are shown in neurological orientation

For the REACT analysis, we employed receptor density maps from the serotonergic and dopaminergic systems (Fig. 1A). Selection of receptors for the primary pharmacology was constrained by availability of PET atlases. The high-resolution in vivo atlases of some serotonergic receptors (Beliveau et al. 2017), including 5HT_1a_, 5HT_1b_ and 5HT_2a_ employed here, are freely available online (https://xtra.nru.dk/FS5ht-atlas/) and were created from molecular and structural high-resolution PET and MRI data of 210 healthy subjects. The D1 receptor atlas was derived from thirteen healthy volunteers using the D1-selective radioligand [^11^C]SCH23390 (Kaller et al. 2017). The D2 receptor atlas was derived using [^18^F]Fallypride PET scans of 6 healthy young adults (Dunn et al. 2009). Voxels within the regions used as references for quantification of the molecular data in the kinetic models for the radioligands were excluded from their respective atlases (which was the cerebellum for all receptor atlases). Finally, all atlases were normalised by scaling image values between 0 and 1 whilst preserving the intensity distribution.

### Receptor-enriched analysis of functional connectivity

The serotonergic and dopaminergic functional systems were estimated with REACT using a two-step regression analysis (Dipasquale et al. 2019) implemented in FSL (fsl_glm command). This is conceptually comparable to the “dual-regression” approach, which is typically used to estimate subject-specific FC maps from template-specific resting-state networks (Nickerson et al. 2017). Within REACT, molecular templates replace the resting-state networks as spatial regressors in the first general linear model. At this stage, both the resting state-fMRI data and the design matrix were also demeaned (–demean option) and masked using a binarised atlas derived from all the molecular data, to restrict the analysis to only those voxels for which receptor density information was available. This stage produced subject-specific time series representing the dominant bold fluctuations within each of these maps. Of note, the five neurotransmitter templates were simultaneously included in the model as previously described in Dipasquale et al. (2019).

The subject-specific time series estimated from this first step was then used as temporal regressors in the second multivariate regression analysis, to estimate the subject-specific target-enriched spatial maps of the BOLD response under LSD and placebo. This was restricted to the grey matter voxels through a binarised mask derived from all participants. Again, both data and design matrix were demeaned (–demean option), with the latter also being normalised to unit standard deviation (–des_norm option). Finally, the subject-specific spatial maps of the BOLD response to LSD and placebo estimated in this step were averaged across runs 1 and 3 for each participant, session and receptor.

### Statistical analysis

A paired-sample *t*-test was computed to test for differences between subject-specific target-enriched spatial maps derived for LSD and placebo conditions. Linear relationships were tested between delta FC maps (LSD minus placebo) and subjective reports of phenomenology, as characterised by the three PCA-derived ASC sub-score as well as the VAS measures. Additional exploratory correlations were tested for the individual ASC sub-scores. All analyses were computed using FSL-Randomise (5000 permutations per test and contrast (Winkler et al. 2014)). Variance smoothing (-v option) was employed at 6 mm for the between conditions *t-*test. Clusters were considered significant if *p*_FWE_ < 0.05, corrected for multiple comparisons using the threshold-free cluster enhancement (-TFCE) option (Smith and Nichols 2009). Mean FC values were extracted for each cluster found to be significantly associated with subjective effects. Finally, a post hoc Pearson’s correlation was computed between mean values extracted from the clusters of altered target-enriched FC in cerebellar vermal lobule X and VAS ratings of ego dissolution and the ASC sub-score for disembodiment.

## Results

### Alterations in receptor-enriched networks (LSD vs placebo)

The REACT-based analysis returned one subject-specific map for each neurotransmitter for each session (LSD and placebo). These target-enriched maps averaged across participants are shown in Fig. 1B.

We found significant increases (*p*_FWE_ < 0.05 TFCE cluster corrected) in FC induced by LSD in the 5HT_1a_-, 5HT_1b_-, 5HT_2a_-, D1- and D2-enriched maps (Fig. 1C). Specifically, 5HT_1a_-enriched maps showed a FC increase in the right lingual gyrus. The 5HT_1b_-enriched maps in bilateral cerebellar hemispheric lobules I–IV, right cerebellar hemispheric lobules V/VI, bilateral thalami, supplementary motor areas, bilateral superior parietal lobules, right mid- and posterior cingulate, bilateral temporal occipital fusiform, parahippocampal, lateral occipital, precuneus and ventromedial prefrontal cortices (PFC) as well as right superior frontal, lingual gyri and pre/post-central gyri. Furthermore, we found an LSD-induced FC increase in the 5HT_2a_-enriched maps localised in the right lateral occipital cortex as well as the right lingual, left paracingulate and bilateral precentral gyri. A FC decrease was also observed for both the 5HT_1b_- and 5HT_2a_-enriched maps in the cerebellar vermal lobule X.

The D1 receptor-enriched maps showed LSD-induced FC increases in the right hippocampus, right thalamus, right superior parietal lobe, left occipital pole, lateral occipital cortices, intracalcarine cortices and the right occipital fusiform, right lingual, middle/superior temporal, angular and pre/post-central gyri. Finally, increased FC under LSD was found for the D2-enriched maps in the right middle/superior temporal gyri as well as the left posterior cingulate, intracalcarine, precuneus and right cuneal cortices. No significant FC decreases were found in the maps enriched by the 5HT_1a_, D1 or D2 receptors.

### Correlation with principal components

The LSD-induced changes in various facets of conscious experience, as measured by the ASC, are reported in detail in Carhart-Harris et al. (2016). Briefly, LSD increased scores in all VAS questions and all sub-scores of the ASC except anxiety.

**Fig. 2:**
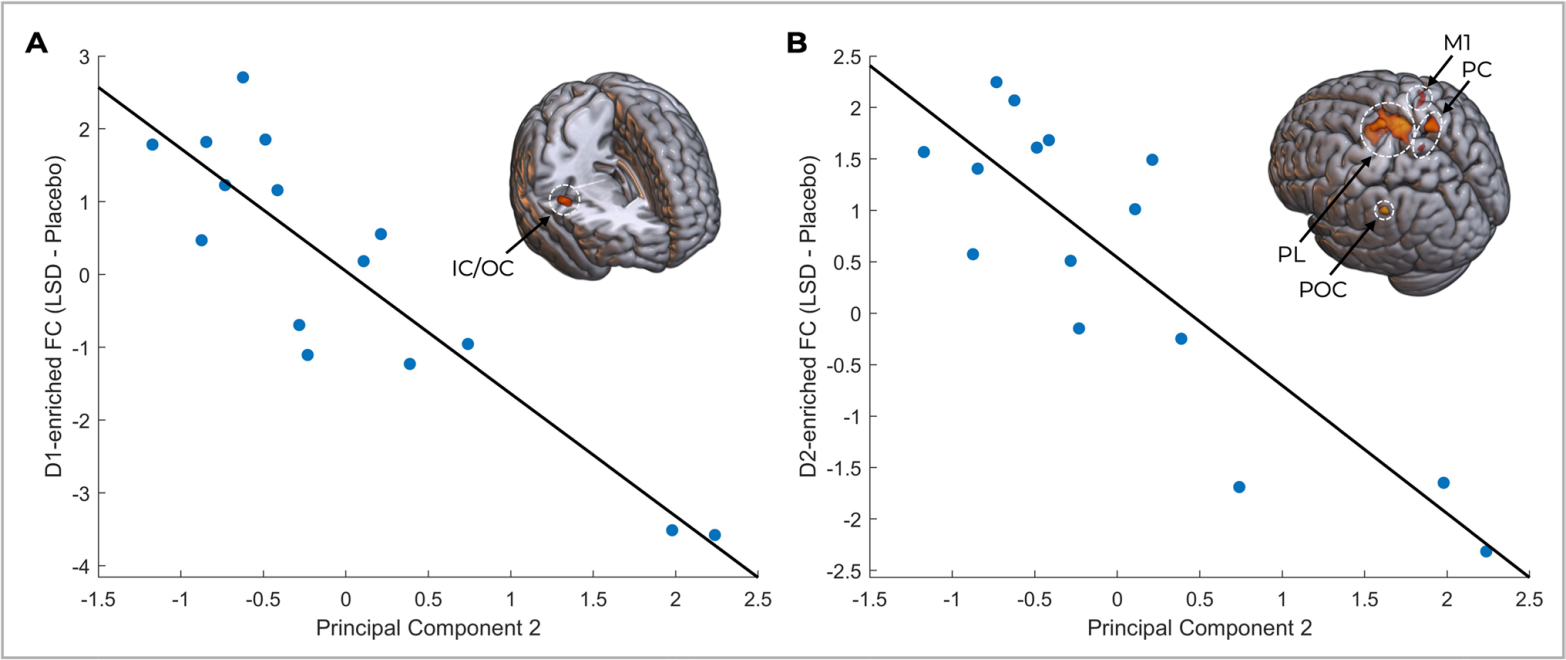
Correlations between receptor-enriched FC (LSD – placebo) and the subjective effects of LSD as measured by the principal components derived from the ASC (**A**) D1- and (**B**) D2-enriched FC showed negative correlations with principal component 2. IC/OC, insular cortex/opercular cortex; PL, paracentral lobule; POC, parietal opercular cortex; M1, primary motor cortex; PC, precuneus cortex

Linear relationships were tested between the target-enriched FC maps and the three principal components derived from the 11 sub-scores of the ASC, which when combined explained 83.1% of the variance. The suitability of the data to PCA was confirmed using Kaiser–Meyer–Olkin Measure of Sampling Adequacy (KMO greater than 0.5) and Bartlett’s test of sphericity (*p* less than 0.05). These components each represent a mixture of differentially loaded aspects of the LSD experience. Principal component one included those with a predominantly positive valence with the strongest loadings for the experience of unity, spiritual experience, blissful state, insightfulness and changed meaning of percepts. Principal component 2 included cognitive aspects with a more negative valence with the strongest loadings for disembodiment as well as impaired control and cognition. Finally, principal component 3 captured mostly perceptual aspects with the strongest loadings for audio-visual synaesthesia, elemental imagery and complex imagery. Two correlations remained significant following TFCE correction; D1-enriched FC in the paracentral lobule (Fig. 2A; *R*^2^ = 0.79) and the D2-enriched FC within the paracentral lobule, parietal opercular cortex, precuneus cortex and primary motor cortex (Fig. 2B; *R*^2^ = 0.73) both with principal component 2. 5HT_2a_-enriched FC extracted from these same clusters did not demonstrate any relationship to disembodiment (*R*^2^ = 0.06 and *R*^2^ = 0.01 respectively). Exploratory relationships with each individual ASC sub-score are reported in the supplementary materials.

### Correlation with VAS questions

**Fig. 3:**
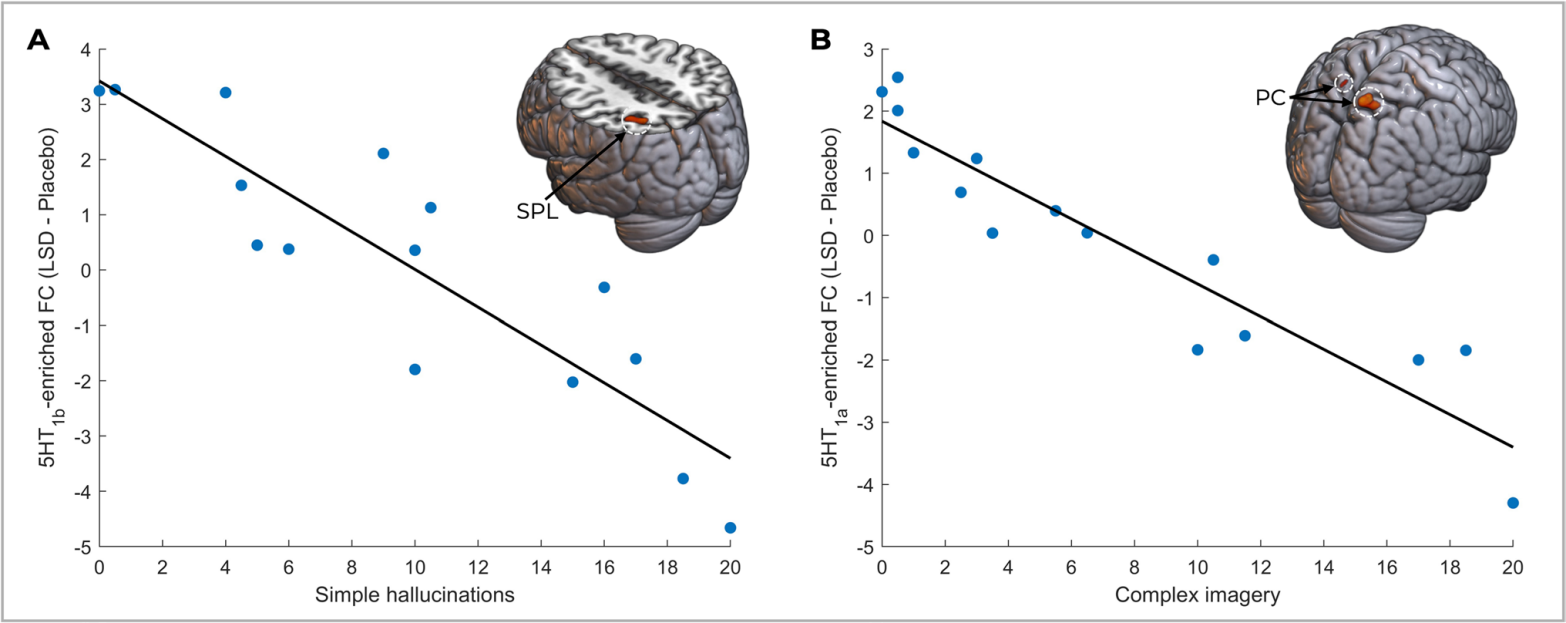
Correlations between receptor-enriched FC (LSD – placebo) and the subjective effects of LSD as measured by VAS questions. **A** 5HT_1b_- and (**B**) 5HT_1a_-enriched FC showed negative correlations with simple hallucinations and complex imagery in the SPL and PC respectively. SPL, superior parietal lobule; PC, precuneus cortex

When correlating the delta target-enriched FC (LSD minus placebo) with VAS responses, only two relationships survived TFCE. Negative correlations were found between 5HT_1b_-enriched FC in the right intraparietal sulcus and simple hallucinations (*R*^2^ = 0.79; Fig. 3A) as well as between 5HT_1a_-enriched FC in the precuneus cortex and complex imagery (*R*^2^ = 0.88; Fig. 3B). 5HT_2a_-enriched FC within the same clusters demonstrated the same relationships (*R*^2^ = 0.66 and *R*^2^ = 0.63 respectively), but these failed to reach significance. Neither D1 nor D2 receptor-enriched FC extracted from these clusters showed relationships with the visual experience. Additionally, based on a post hoc hypothesis, correlation analyses revealed that both FC maps enriched by 5HT_1b_ within cerebellar lobule X (*R*^2^ = 0.20) and 5HT_2a_ (*R*^2^ = 0.16) correlated with reported ego dissolution, but not disembodiment.

## Discussion

The present study offers new insights into the pharmacodynamic response of the brain under LSD. FC within 5HT_1a_**-**, 5HT_1b_**-**, 5HT_2a_**-**, D1**-** and D2**-**enriched maps showed significant differences following administration of LSD as compared to placebo. Moreover, target-enriched FC demonstrated various relationships to the subjective effects of LSD on conscious experience with serotonergic systems being predominantly related to perceptual effects whilst dopaminergic systems were broadly linked with aspects of perceived selfhood and cognition. Various neurobiological theories of the psychedelic state have been put forth including the cortico-striatal thalamocortical (CSTC) (Vollenweider and Geyer 2001; Vollenweider and Preller 2020) and relaxed beliefs under psychedelics (REBUS) models (Carhart-Harris and Friston 2019). These propose reduced thalamic gating that leads to increased information flow to the cortex and reduced precision of high-level priors respectively. Multiple components of CSTC loops are under the modulatory influence of both the serotonergic and dopaminergic systems (Swerdlow et al. 2001), and two imaging studies have described increased thalamic FC under LSD, especially with sensory and somato-motor regions (Müller et al. 2017; Preller et al. 2018). This broadly aligns with our findings of increased 5HT_1a_-enriched FC in the thalamus as well as various regions of the sensory and somato-motor cortex across the receptor systems. Moreover, a dynamic causal modelling study evaluating the CSTC model found that the striatum reduces its inhibitory control over the thalamus under LSD, but also that this is not blocked by ketanserin, suggesting a non-5HT_2a_-mediated mechanism (Preller et al. 2019). This may be driven by dopamine or other serotonergic receptors, though the current analyses did not show altered striatal FC, possibly due to a lack of power. Additionally, the REBUS model places a strong emphasis on the 5HT_2a_ receptor, claiming its dense expression in high-level cortical regions provides an anatomical basis for disruption high up in the brain’s functional hierarchy (Carhart-Harris and Friston 2019). However, the different serotonin receptor-enriched networks identified here are extremely similar and derived based upon their spatial distributions, highlighting the need to also consider the pleiotropic mechanisms of these receptors.

### The serotonergic system

In accordance with its primarily serotonergic mechanism of action, we found widespread LSD-induced FC differences in functional systems enriched by different serotonergic receptors. To date, there remains a lack of research investigating non-5HT_2a_-mediated mechanisms. Global brain connectivity (GBC) under LSD has been described to significantly spatially correlate with the distribution of the 5HT_2a_ receptor, but not the 5HT_1a_ and 5HT_1b_ receptors (Tagliazucchi et al. 2016). Conversely, we found significant differences in 5HT_1a_- and 5HT_1b_-enriched FC. GBC has also been investigated under LSD compared to LSD alongside ketanserin in relation to the expression of candidate serotonergic and dopaminergic receptors, as quantified within the Allen Human Brain Atlas (AHBA) (Preller et al. 2018). However, transcriptomic data is available from a single hemisphere of only 6 participants and whilst the 5HT_1a_ receptor atlas employed here shows excellent correlations with the AHBA data, the 5HT_2a_ and 5HT_1b_ receptors showed only weak correlations cortically and no significant relationship subcortically (Beliveau et al. 2017). As such, differences in our findings to prior work likely result from differences in the distributions of PET (Tagliazucchi et al. 2016) and transcriptomic data (Preller et al. 2018). Here, we employed high-resolution atlases derived from 210 individuals (Beliveau et al. 2017), which may allow for a more careful delineation of these different serotonergic receptor systems. However, whilst these findings may represent novel contributions of the 5HT_1a_ and 5HT_1b_ receptors, the PET data and receptor-enriched networks for the different serotonergic receptors demonstrate strong overlap. Furthermore, serotonin selective reuptake inhibitors produce similar network alterations as LSD without producing profound effects on consciousness (Klaassens et al. 2015). As such, these results should be interpreted with caution. 5HT_1a_ agonism reportedly diminished visual effects of psilocybin and 5HT_1a_ antagonism bolstered the effects of N,N-dimethyltryptamine (Strassman 1995; Pokorny et al. 2016). Similar studies employing pharmacological manipulation of 5HT_1a/b_ under LSD will be crucial to causally investigate their contribution to its effects on conscious experience.

The 5HT_1a_- and 5HT_1b_-enriched FC in the superior parietal lobules and precuneus correlated with simple hallucinations and complex imagery respectively. The same relationships were also present for the 5HT_2a_ receptor, though these failed to reach significance, suggesting that these receptors may act in concert, with visual experiences relating to the functions of the serotonin system more broadly. LSD-induced visual hallucinations have been described to correlate with FC in the thalamus and right fusiform, purportedly reflecting altered thalamic gating of percepts (Müller et al. 2017). Similarly, LSD elicits responses in the primary and tertiary visual cortex during the eyes-closed resting state similar to those seen under visual stimulation (Müller et al. 2017) and the primary visual cortex shows widespread increased FC, including to the precuneus (Carhart-Harris et al. 2016). The precuneus shows increased blood flow during memory-related visual imagery, possibly reflecting the incorporation of autobiographical information into percepts (Fletcher et al. 1995). Psychedelic-related visual experiences are frequently reported as deeply profound and having personalised meaning (Kometer et al. 2016). As such, they not only alter the visual percept, but also the relationship between the viewer and the percept, and this may involve the precuneus given its longstanding associations to visuo-spatial imagery, episodic memory retrieval, and self-referential processing (Cavanna and Trimble 2006). Finally, the precuneus and superior parietal cortex have been implicated in clinical hallucinations (Carter and Ffytche 2015), and these have also been linked to the density of the 5HT_1a_ and 5HT_2a_ receptors (Vignando et al. 2022). Moreover, these regions constitute part of the dorsal attentional network, in which dysfunction is central to transdiagnostic attentional theories of visual hallucination (Shine et al. 2014; Lawn and Ffytche 2020). Intracranial stimulation in this area can also evoke illusionary movement, providing clearer causal evidence for its capacity to generate aberrant percepts (Perumal et al. 2014). Thus, the correlations between serotonergic FC and visual experience observed here may constitute altered higher-level integration of visual and autobiographical information, with potential contributions of altered bottom-up information flow, thalamocortical connectivity and attentional networks.

The only region showing an LSD-induced FC decrease was the cerebellar vermal lobule X, for both the 5HT_1b_- and 5HT_2a_-enriched maps. This region is thought to play a crucial role in the representation of self-motion through the processing of spatial and temporal vestibular information (Cullen 2011; Baumann et al. 2015). Lesions of this area diminish the perception of self-motion (Bronstein et al. 2008; Bertolini et al. 2012) and visually induced illusions of self-motion preferentially activate it (Kleinschmidt et al. 2002; Bense et al. 2006). The sense of self is dependent on multisensory integration of not only visual and somatosensory, but also vestibular information (Lenggenhager et al. 2006; Pfeiffer et al. 2013; Limanowski 2014; Qin et al. 2020). We found negative correlations in post hoc analyses for both the 5HT_1b_- and 5HT_2a_-enriched FC in this region with VAS ratings of ego dissolution, but not ASC ratings of disembodiment. The relatively low variance explained is in accordance with a minor contribution of vestibular processing to perceived selfhood. However, it is unclear why ego dissolution demonstrated this relationship whilst disembodiment did not. These probe different facets of autonoetic consciousness (integrated sense of self and body ownership respectively), and the ASC ratings were conducted retrospectively, whilst the VAS ratings followed each resting-state run. We speculate that LSD-induced altered serotonergic FC in the vestibulocerebellum relates to altered integration of vestibular information into multisensory mechanisms underlying the sense of self-motion and that this is a minor facet of ego dissolution induced by LSD.

### The dopaminergic system

Robust differences in D1- and D2-enriched FC were observed between LSD and placebo conditions. Both D1- and D2-enriched FC in a set of somato-motor, superior parietal and insular/opercular regions showed negative correlations with principal component 2, which had loadings from cognitive aspects of predominantly negative valence (disembodiment as well as impaired control and cognition). Interestingly, many questions constituting impaired control and cognition also relate to perceived selfhood, e.g. “I felt like a marionette”, “I felt isolated from everything and everyone” and “I had the feeling that I no longer had a will of my own”. As such, these relationships are likely predominantly driven by experiences of selfhood, though more specific confirmatory approaches are required to determine this.

The right posterior insula has long been implicated in subjective body ownership (Tsakiris et al. 2007); strokes affecting this region can produce anosognosia or somatoparaphrenia, in which individuals deny impairment of sensory or motor function and assert that a part of their body belongs to someone else respectively (Karnath et al. 2005; Baier and Karnath 2008; Karnath and Baier 2010b, a). Our findings also align with several meta-analyses of body ownership, agency, multisensory integration and self-awareness, as well as body ownership (Grivaz et al. 2017; Seghezzi et al. 2019; Salvato et al. 2020), which broadly implicate regions including the insula and superior parietal cortices as well as somato-motor and temporoparietal areas. Additionally, integration of interoceptive and exteroceptive signals has been suggested to engage a pre-motor cortex-intraparietal sulcus-insula network (Park and Blanke 2019). LSD-induced disembodiment has also previously been described to correlate with GBC in the somato-motor network (Preller et al. 2018) as well as bilateral insular cortex and angular gyrus (Tagliazucchi et al. 2016). Although some studies have investigated its contribution to conscious self-monitoring (Joensson et al. 2015) and self-awareness (Lou et al. 2020), the precise role of dopamine in these processes remains unclear. However, a pharmacologically induced increase in synaptic dopamine has been reported to decrease the sense of ownership of ones’ own hand during the rubber hand illusion (Albrecht et al. 2011). Kappa opioid receptor agonists like salvinorin-A (the psychoactive component of *Salvia divinorum* (Roth et al. 2002; Stiefel et al. 2014) also act partly through modulating dopamine levels (Ebner et al. 2010), though directionality and dose-dependency remain highly equivocal. Critically, whilst it differs from classic serotonergic psychedelics in not acting on the 5HT_2a_ receptor, it does produce profound somatosensory changes, disembodiment, as well as perturbations of cognition which may be partly mediated by functional dopaminergic circuits (Addy 2012; Addy et al. 2015). We suggest that somato-motor, parietal, opercular and insular regions of the D1- and D2-enriched FC correlating with principal component 2 here constitute part of a broader functional network, are associated with modulatory effects of dopamine and contribute to LSD-induced experiences of disembodiment and impaired cognition.

It has been reported that neural activity associated with LSD alongside ketanserin varies between 75 and 300 min post-dose, suggesting a potentially phasic involvement of different neurotransmitter systems being described within the preclinical literature (Marona-Lewicka et al. 2005; Marona-Lewicka and Nichols 2007; Preller et al. 2018). Specifically, it has been suggested that following an initial 5HT_2a_-driven phase, there is a subsequent phase for which dopamine receptor activation is more important. Of note, Minuzzi et al. showed that LSD had an unusual effect on D2/D3 receptor binding measured using [^11^C]raclopride PET; progressive displacement of the ligand which only peaked 240 min post-administration (Minuzzi et al. 2005). The authors suggested that this may reflect a 5HT_2_-mediated sensitisation of D2/D3 receptors, a mechanism also postulated elsewhere (Marona-Lewicka and Nichols 1997; Nichols 2016). Crucially, this may mean that experiments blocking the action of LSD on the 5HT_2a_ receptor utilising ketanserin are also preventing dopamine receptor sensitisation and thus masking the role of dopamine in the effects of LSD (Preller et al. 2018). Furthermore, resting-state scans here were undertaken roughly 70 min post-dosing. Whilst potential differences in these time-dependent effects and phases between animals and humans have yet to be elucidated, our results may be related to an earlier serotonergic phase. Thus, despite the robust differences in D1 and D2 receptor-enriched FC observed, these findings may be even stronger with data acquired later in the LSD experience with resultant changes in experiential correlates also likely to shift as a function of time. A better characterisation of the contribution of these systems and delineating of these phases, possibly employing pharmacological manipulation of dopamine, certainly warrants further investigation.

### Limitations

To the best of our knowledge, this is the first study to examine the modulation of receptor-enriched networks under LSD as well as correlate subject-specific FC changes with subjective effects. However, we should highlight some limitations of the present study. First, the molecular density information of the PET atlases used for REACT was derived from independent datasets of healthy subjects, assuming that the distribution of those neurotransmitters in the brain of healthy populations is comparable. Secondly, we acknowledge that the sample contained a significantly greater number of female participants and so we cannot exclude the potential for sex-based differences in pharmacodynamics. We encourage consideration of substratification and assessment of sex differences in future investigations in suitably powered cohorts. Finally, the sample size employed here is modest, limiting the power to detect relationships within a voxelwise framework. Correlations between target-enriched FC and subjective effects were generally strong, but larger samples may be required for these to reach significance within a voxelwise regression framework which, unlike the main effects, does not benefit from the power advantages of a within-subjects design.

## Conclusion

LSD has a complex pharmacology that interacts with diverse neural systems. Despite prevailing 5HT_2a_-centric mechanistic theories of LSD, here we characterised differences in FC associated with the 5HT_1a_, 5HT_1b_, D1 and D2 receptors as well as their experiential correlates. The dopaminergic system was associated with LSD effects on perceived selfhood and cognition, whilst serotonergic systems were involved in hallucinations. Crucially, these correlations demonstrated a double dissociation with the different receptors showing the same relationship within, but not between, the serotonergic and dopaminergic systems. The effects of LSD on dopaminergic systems may also be conditional on 5HT_2a_ mechanisms, which warrants subsequent direct investigation. It will be imperative to replicate these exploratory findings in a separate dataset better powered to assess relationships with traditional psychometry and novel outcome measures relevant to clinical populations as well as potentially employ mediation analyses to probe interactions between systems. Pharmacological manipulation of additional receptor systems alongside the administration of LSD provides an enticing avenue for a more definitive mechanistic elucidation, which may lead to translational and therapeutic benefit in the longer term.

## Supplementary Information

Below is the link to the electronic supplementary material.

Supplementary file1 (DOCX 1257 KB)

## Declarations

### Disclaimer

The views expressed are those of the authors and not necessarily those of the NHS, the NIHR or the Department of Health and Social Care.

## References

[CR1] Addy PH (2012). Acute and post-acute behavioral and psychological effects of salvinorin A in humans. Psychopharmacology.

[CR2] Addy PH, Garcia-Romeu A, Metzger M, Wade J (2015). The subjective experience of acute, experimentally-induced Salvia divinorum inebriation. J Psychopharmacol.

[CR3] Albrecht MA, Martin-Iverson MT, Price G (2011). Dexamphetamine effects on separate constructs in the rubber hand illusion test. Psychopharmacology.

[CR4] Aston-Jones G, Cohen JD (2005). An integrative theory of locus coeruleus-norepinephrine function: adaptive gain and optimal performance. Annu Rev Neurosci.

[CR5] Avery MC, Krichmar JL (2017). Neuromodulatory systems and their interactions: a review of models, theories, and experiments. Frontiers in Neural Circuits.

[CR6] Baier B, Karnath HO (2008). Tight link between our sense of limb ownership and self-awareness of actions. Stroke.

[CR7] Baumann O, Borra RJ, Bower JM (2015). Consensus paper: the role of the cerebellum in perceptual processes. The Cerebellum.

[CR8] Beliveau V, Ganz M, Feng L (2017). A high-resolution in vivo atlas of the human brain’s serotonin system. J Neurosci.

[CR9] Bense S, Janusch B, Vucurevic G (2006). Brainstem and cerebellar fMRI-activation during horizontal and vertical optokinetic stimulation. Exp Brain Res.

[CR10] Bertolini G, Ramat S, Bockisch CJ, et al (2012) Is vestibular self-motion perception controlled by the velocity storage? Insights from patients with chronic degeneration of the vestibulo-cerebellum. PLoS ONE 7:.10.1371/journal.pone.0036763 10.1371/journal.pone.0036763 PMC3376140 22719833

[CR11] Bronstein AM, Grunfeld EA, Faldon M, Okada T (2008). Reduced self-motion perception in patients with midline cerebellar lesions. NeuroReport.

[CR12] Carhart-Harris RL, Friston KJ (2019). REBUS and the anarchic brain: toward a unified model of the brain action of psychedelics. Pharmacol Rev.

[CR13] Carhart-Harris RL, Muthukumaraswamy S, Roseman L (2016). Neural correlates of the LSD experience revealed by multimodal neuroimaging. Proc Natl Acad Sci USA.

[CR14] Carter R, Ffytche DH (2015). On visual hallucinations and cortical networks: a trans-diagnostic review. J Neurol.

[CR15] Cavanna AE, Trimble MR (2006). The precuneus: a review of its functional anatomy and behavioural correlates. Brain.

[CR16] Cullen KE (2011). The neural encoding of self-motion. Curr Opin Neurobiol.

[CR17] de Gregorio D, Comai S, Posa L, Gobbi G (2016a) d-Lysergic acid diethylamide (LSD) as a model of psychosis: mechanism of action and pharmacology. Int J Mol Sci 17:1953 10.3390/ijms17111953 PMC5133947 27886063

[CR18] de Gregorio D, Posa L, Ochoa-Sanchez R et al (2016b) The hallucinogen d-lysergic diethylamide (LSD) decreases dopamine firing activity through 5-HT1A, D2 and TAAR1 receptors. Pharmacol Res 113:81–91. 10.1016/J.PHRS.2016.08.022 10.1016/j.phrs.2016.08.022 27544651

[CR19] de Gregorio D, Aguilar-Valles A, Preller KH (2021). Hallucinogens in mental health: preclinical and clinical studies on LSD. Psilocybin, MDMA, and Ketamine.

[CR20] Dipasquale O, Selvaggi P, Veronese M (2019). Receptor-enriched analysis of functional connectivity by targets (REACT): a novel, multimodal analytical approach informed by PET to study the pharmacodynamic response of the brain under MDMA. Neuroimage.

[CR21] Dunn J, Stone J, Cleij M (2009). Differential occupancy of striatal versus extrastriatal dopamine D2/D3 receptors by the typical antipsychotic haloperidol in man measured using [18F]-Fallypride PET. Neuroimage.

[CR22] Ebner SR, Roitman MF, Potter DN (2010). Depressive-like effects of the kappa opioid receptor agonist salvinorin A are associated with decreased phasic dopamine release in the nucleus accumbens. Psychopharmacology.

[CR23] Fletcher PC, Frith CD, Baker SC (1995). The mind’s eye—precuneus activation in memory-related imagery. Neuroimage.

[CR24] Grivaz P, Blanke O, Serino A (2017). Common and distinct brain regions processing multisensory bodily signals for peripersonal space and body ownership. Neuroimage.

[CR25] Hofmann A (1979). How LSD originated†. J Psychoactive Drugs.

[CR26] Jobst BM, Atasoy S, Ponce-Alvarez A (2021). Increased sensitivity to strong perturbations in a whole-brain model of LSD. Neuroimage.

[CR27] Joensson M, Thomsen KR, Andersen LM (2015). Making sense: dopamine activates conscious self-monitoring through medial prefrontal cortex. Hum Brain Mapp.

[CR28] Kaller S, Rullmann M, Patt M (2017). Test–retest measurements of dopamine D1-type receptors using simultaneous PET/MRI imaging. Eur J Nucl Med Mol Imaging.

[CR29] Karnath HO, Baier B (2010). Right insula for our sense of limb ownership and self-awareness of actions. Brain Struct Funct.

[CR30] Karnath HO, Baier B, Nag̈ele T (2005). Awareness of the functioning of one’s own limbs mediated by the insular cortex?. J Neurosci.

[CR31] Karnath H-O, Baier B (2010b) Anosognosia for hemiparesis and hemiplegia: disturbed sense of agency and body ownership. The Study of Anosognosia 39–62

[CR32] Kast E, Analgesia VC (1964). Study of lysergic acid diethylamide as an analgesic agent. Anesthesia.

[CR33] Klaassens BL, van Gorsel HC, Khalili-Mahani N (2015). Single-dose serotonergic stimulation shows widespread effects on functional brain connectivity. Neuroimage.

[CR34] Kleinschmidt A, Thilo KV, Büchel C (2002). Neural correlates of visual-motion perception as object- or self-motion. NeuroImage.

[CR35] Kometer M, Vollenweider FX, Kometer M, Vollenweider FX (2016). Serotonergic hallucinogen-induced visual perceptual alterations. Curr Top Behav Neurosci.

[CR36] Kraehenmann R, Pokorny D, Vollenweider L (2017). Dreamlike effects of LSD on waking imagery in humans depend on serotonin 2A receptor activation. Psychopharmacology.

[CR37] Krebs TS, Johansen PØR (2012). Lysergic acid diethylamide (LSD) for alcoholism: meta-analysis of randomized controlled trials. J Psychopharmacol.

[CR38] Lawn T, Ffytche D (2020). Cerebellar correlates of visual hallucinations in Parkinson’s disease and Charles Bonnet Syndrome. Cortex.

[CR39] Lenggenhager B, Smith ST, Blanke O (2006). Functional and neural mechanisms of embodiment: importance of the vestibular system and the temporal parietal junction. Rev Neurosci.

[CR40] Limanowski J (2014) What can body ownership illusions tell us about minimal phenomenal selfhood? Frontiers in Human Neuroscience 8:.10.3389/FNHUM.2014.00946/FULL 10.3389/fnhum.2014.00946 PMC4241829 25505398

[CR41] Lou HC, Rømer Thomsen K, Changeux J-P (2020). The molecular organization of self-awareness: paralimbic dopamine-GABA interaction. Front Syst Neurosci.

[CR42] Luppi AI, Carhart-Harris RL, Roseman L, et al (2021) LSD alters dynamic integration and segregation in the human brain. NeuroImage 227:.10.1016/j.neuroimage.2020.117653 10.1016/j.neuroimage.2020.117653 PMC7896102 33338615

[CR43] Marder E (2012). Neuromodulation of neuronal circuits: back to the future. Neuron.

[CR44] Marona-Lewicka D, Nichols DE (1997). 5-HT(2A)/(2C) receptor agonists potentiate the discriminative cue of (+)-amphetamine in the rat. Neuropharmacology.

[CR45] Marona-Lewicka D, Nichols DE (2007). Further evidence that the delayed temporal dopaminergic effects of LSD are mediated by a mechanism different than the first temporal phase of action. Pharmacol Biochem Behav.

[CR46] Marona-Lewicka D, Kurrasch-Orbaugh DM, Selken JR (2002). Re-evaluation of lisuride pharmacology: 5-hydroxytryptamine1A receptor-mediated behavioral effects overlap its other properties in rats. Psychopharmacology.

[CR47] Marona-Lewicka D, Thisted RA, Nichols DE (2005). Distinct temporal phases in the behavioral pharmacology of LSD: dopamine D2 receptor-mediated effects in the rat and implications for psychosis. Psychopharmacology.

[CR48] Minuzzi L, Nomikos GG, Wade MR (2005). Interaction between LSD and dopamine D2/3 binding sites in pig brain. Synapse.

[CR49] Müller F, Lenz C, Dolder P (2017). Increased thalamic resting-state connectivity as a core driver of LSD-induced hallucinations. Acta Psychiatr Scand.

[CR50] Nichols DE (2004). Hallucinogens. Pharmacol Ther.

[CR51] Nichols DE (2016). Psychedelics. Pharmacol Rev.

[CR52] Nickerson LD, Smith SM, Öngür D, Beckmann CF (2017) Using dual regression to investigate network shape and amplitude in functional connectivity analyses. Frontiers in Neuroscience 11:.10.3389/fnins.2017.00115 10.3389/fnins.2017.00115 PMC5346569 28348512

[CR53] Nutt D (2015) Illegal drugs laws: clearing a 50-year-old obstacle to research. PLoS Biology 13:.10.1371/journal.pbio.1002047 10.1371/journal.pbio.1002047 PMC4307971 25625189

[CR54] Pahnke WN, Kurland AA, Goodman LE, Richards WA (1969). LSD-assisted psychotherapy with terminal cancer patients. Curr Psychiatr Ther.

[CR55] Pahnke WN, Kurland AA, Unger S (1970). The experimental use of psychedelic (LSD) psychotherapy. JAMA: The Journal of the American Medical Association.

[CR56] Park HD, Blanke O (2019). Coupling inner and outer body for self-consciousness. Trends Cogn Sci.

[CR57] Passie T, Halpern JH, Stichtenoth DO (2008). The pharmacology of lysergic acid diethylamide: a review. CNS Neurosci Ther.

[CR58] Perumal MB, Chinnasami S, Shah A (2014). Epileptic kinetopsia localizes to superior parietal lobule and intraparietal sulcus. Neurology.

[CR59] Pfeiffer C, Lopez C, Schmutz V, et al (2013) Multisensory origin of the subjective first-person perspective: visual, tactile, and vestibular mechanisms. PLoS ONE 8:.10.1371/JOURNAL.PONE.0061751 10.1371/journal.pone.0061751 PMC3632612 23630611

[CR60] Pokorny T, Preller KH, Kraehenmann R, Vollenweider FX (2016). Modulatory effect of the 5-HT1A agonist buspirone and the mixed non-hallucinogenic 5-HT1A/2A agonist ergotamine on psilocybin-induced psychedelic experience. Eur Neuropsychopharmacol.

[CR61] Preller KH, Herdener M, Pokorny T (2017). The fabric of meaning and subjective effects in LSD-induced states depend on serotonin 2A receptor activation. Curr Biol.

[CR62] Preller KH, Razi A, Zeidman P (2019). Effective connectivity changes in LSD-induced altered states of consciousness in humans. Proc Natl Acad Sci USA.

[CR63] Preller KH, Burt JB, Ji JL, et al (2018) Changes in global and thalamic brain connectivity in LSD-induced altered states of consciousness are attributable to the 5-HT2A receptor. eLife 7:. 10.7554/eLife.35082 10.7554/eLife.35082 PMC6202055 30355445

[CR64] Qin P, Wang M, Northoff G (2020). Linking bodily, environmental and mental states in the self—a three-level model based on a meta-analysis. Neurosci Biobehav Rev.

[CR65] Roth BL, Baner K, Westkaemper R (2002). Salvinorin A: a potent naturally occurring nonnitrogenous κ opioid selective agonist. Proc Natl Acad Sci USA.

[CR66] Salvato G, Richter F, Sedeño L (2020). Building the bodily self-awareness: evidence for the convergence between interoceptive and exteroceptive information in a multilevel kernel density analysis study. Hum Brain Mapp.

[CR67] Seghezzi S, Giannini G, Zapparoli L (2019). Neurofunctional correlates of body-ownership and sense of agency: a meta-analytical account of self-consciousness. Cortex.

[CR68] Shine JM, O’Callaghan C, Halliday GM, Lewis SJG (2014). Tricks of the mind: visual hallucinations as disorders of attention. Prog Neurobiol.

[CR69] Shine JM, Breakspear M, Bell PT (2019). Human cognition involves the dynamic integration of neural activity and neuromodulatory systems. Nat Neurosci.

[CR70] Smith SM, Nichols TE (2009). Threshold-free cluster enhancement: addressing problems of smoothing, threshold dependence and localisation in cluster inference. Neuroimage.

[CR71] Stiefel KM, Merrifield A, Holcombe AO (2014). The claustrum’s proposed role in consciousness is supported by the effect and target localization of Salvia divinorum. Front Integr Neurosci.

[CR72] Strassman RJ (1995). Human psychopharmacology of N, N-dimethyltryptamine. Behav Brain Res.

[CR73] Studerus E, Gamma A, Vollenweider FX (2010). Psychometric evaluation of the Altered States of Consciousness Rating Scale (OAV). PLoS ONE.

[CR74] Swerdlow NR, Geyer MA, Braff DL (2001). Neural circuit regulation of prepulse inhibition of startle in the rat: current knowledge and future challenges. Psychopharmacology.

[CR75] Tagliazucchi E, Roseman L, Kaelen M (2016). Increased global functional connectivity correlates with LSD-induced ego dissolution. Curr Biol.

[CR76] Tsakiris M, Schütz-Bosbach S, Gallagher S (2007). On agency and body-ownership: phenomenological and neurocognitive reflections. Conscious Cogn.

[CR77] Vignando M, Ffytche D, Lewis SJG (2022). Mapping brain structural differences and neuroreceptor correlates in Parkinson’s disease visual hallucinations. Nat Commun.

[CR78] Vollenweider FX, Geyer MA (2001). A systems model of altered consciousness: integrating natural and drug-induced psychoses. Brain Res Bull.

[CR79] Vollenweider FX, Preller KH (2020). Psychedelic drugs: neurobiology and potential for treatment of psychiatric disorders. Nat Rev Neurosci.

[CR80] Winkler AM, Ridgway GR, Webster MA (2014). Permutation inference for the general linear model. Neuroimage.

