# Supplementary material for: Differential contributions of serotonergic and dopaminergic functional connectivity to the phenomenology of LSD
Source: Psychopharmacology (Berl). 2022 Mar 24;239(6):1797–808. doi: 10.1007/s00213-022-06117-5 (PMC9166846; doi:10.1007/s00213-022-06117-5)
Supplement: Supplementary file 1 — Supplementary file1 (DOCX 1257 KB) [file 213_2022_6117_MOESM1_ESM.docx]

**Differential Contributions of Serotoninergic and Dopaminergic Functional Connectivity to the Phenomenology of LSD**

*Timothy Lawn (timothy.lawn@kcl.ac.uk) MSc^1^, Ottavia Dipasquale PhD^1^, Alexandros Vamvakas MSc^1,2^, Ioannis Tsougos PhD^1,2^, Mitul A. Mehta PhD^1^, Matthew A. Howard PhD^1^*

*^1^* *Department of Neuroimaging, Institute of Psychiatry, Psychology and Neuroscience, King’s college London, London, UK*

^2^ *School of Medicine, University of Thessaly, Volos, Greece*

***Supplementary Information***


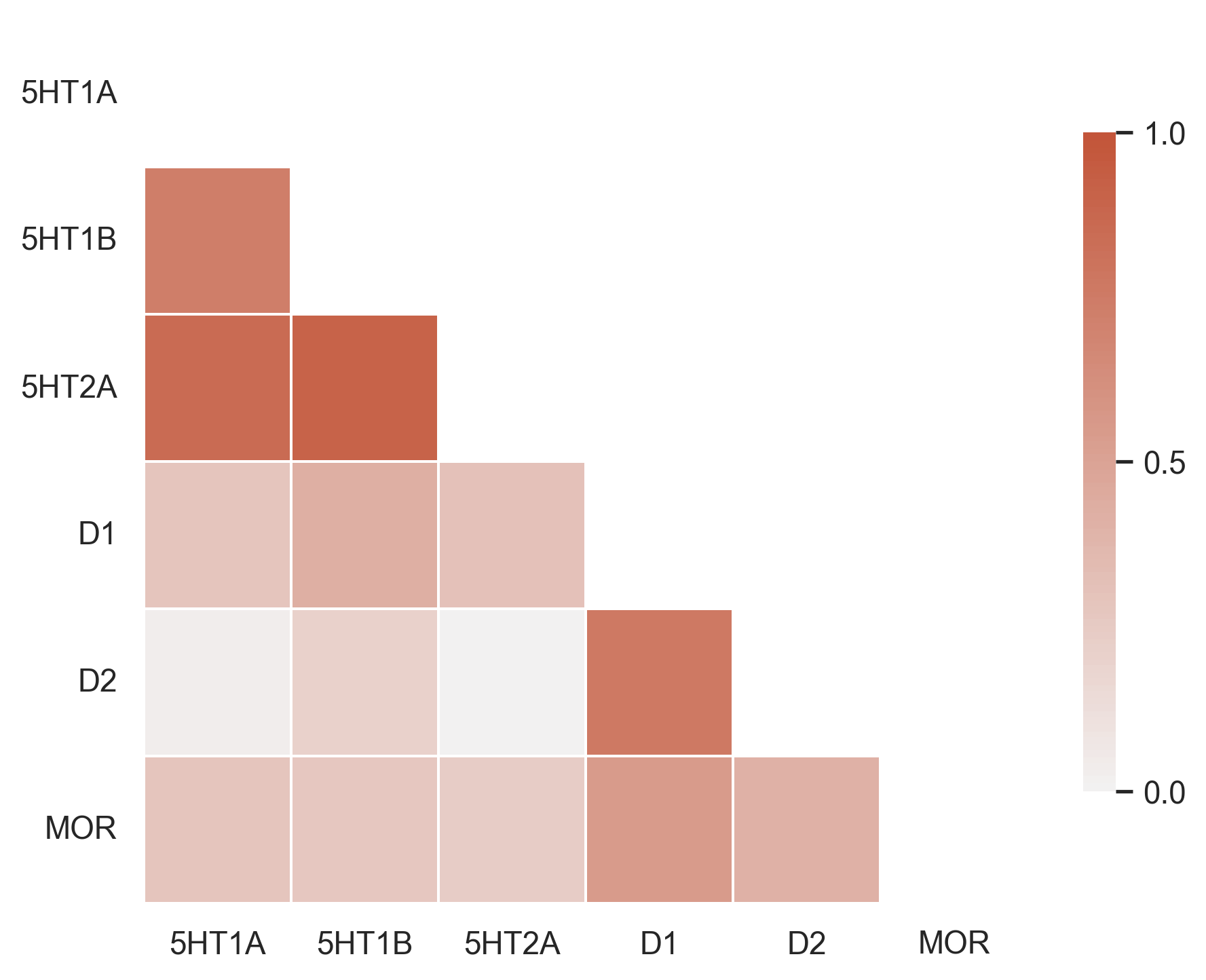


**SI fig.1** Bivariate spatial correlation coefficients between each of the PET receptor systems

**SI fig.2** correlations between receptor enriched FC the subjective effects of LSD as measured by the individual ASC sub-scores. Several correlations were found to be significant following correction for multiple comparisons across voxels using TFCE. Serotonergic FC showed relationships with altered meaning of percepts (A/B/D) and blissful state (C). Dopaminergic FC showed relationships with spiritual experience and anxiety (E/F), and with disembodiment (G/H). *SPL; Superior parietal lobule. SMG; Supramarginal gyrus. IPL; Inferior parietal lobule. ACC; Anterior cingulate cortex. vmPFC; Ventromedial prefrontal cortex. SFG; Superior frontal gyrus. PCS; Posterior cingulate sulcus. PI; Posterior Insula. PL; Paracentral lobule.*


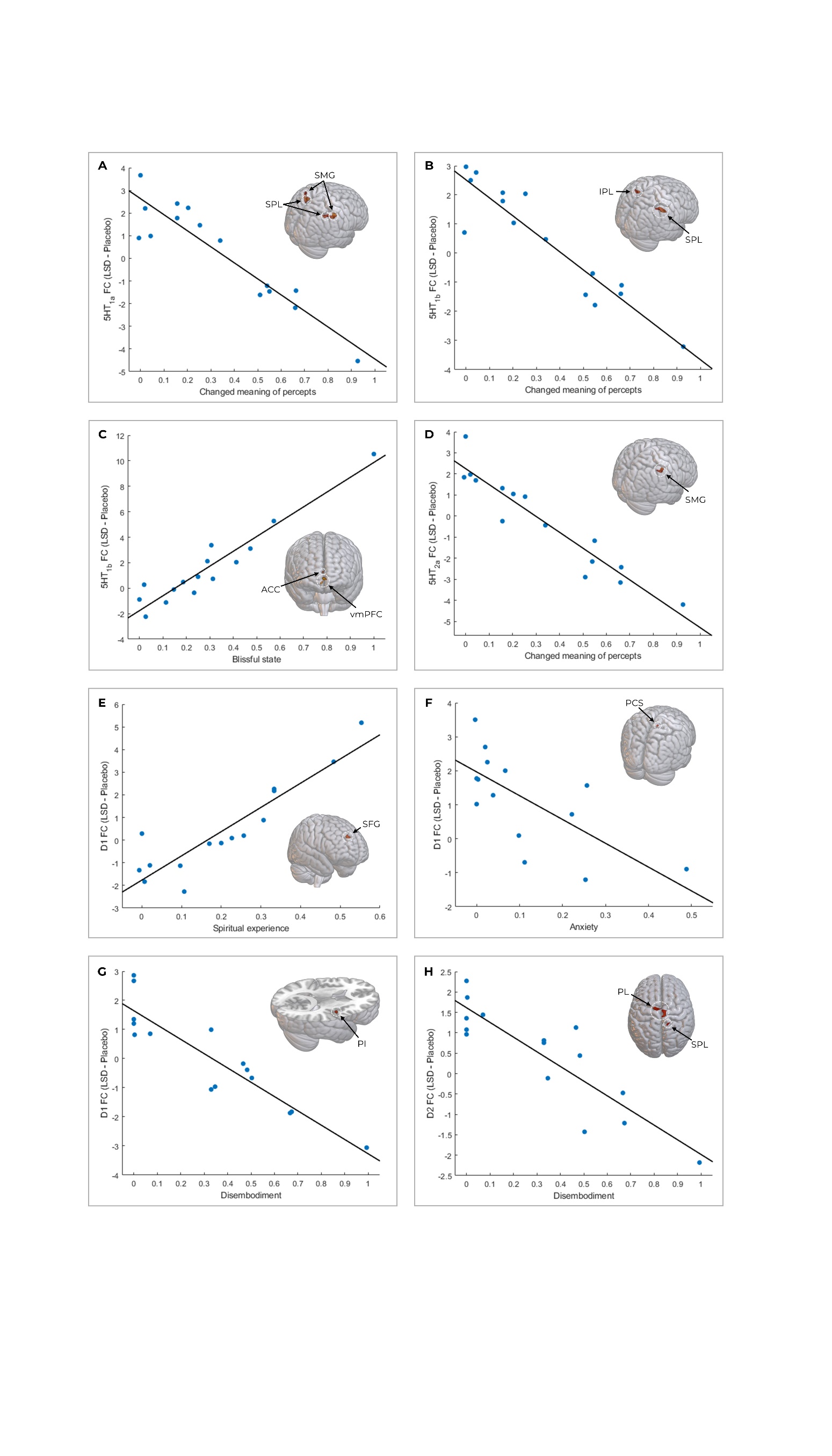


| ***Supplementary table 1****: correlations between receptor enriched FC and principal components as well as VAS scores. Results are shown TFCE corrected at p_FWE_ < 0.1 to additionally show clusters nearing significance. Significant clusters are emboldened.* | | | | | |
| --- | --- | --- | --- | --- | --- |
|  |  |  | MNI co-ordinates | | |
|  | Voxels | *p* value | MAX X | MAX Y | MAX Z |
| D1 enriched FC - Principal component 2 - Negative correlation | **247** | **0.045** | **-6** | **-14** | **68** |
|  | **191** | **0.026** | **48** | **-8** | **10** |
|  | **44** | **0.049** | **-40** | **-38** | **18** |
|  | 5 | 0.098 | 10 | -14 | 50 |
|  |  |  |  |  |  |
| D2 enriched FC - Principal component 2 - Negative correlation | **1998** | **0.011** | **8** | **-24** | **58** |
|  | **458** | **0.042** | **38** | **-18** | **56** |
|  | **72** | **0.007** | **-40** | **-38** | **18** |
|  | **68** | **0.048** | **34** | **-8** | **0** |
|  | 17 | 0.094 | 64 | 6 | 14 |
|  | 13 | 0.09 | 66 | -4 | 2 |
|  | 10 | 0.086 | 50 | -6 | 10 |
|  |  |  |  |  |  |
| D1 enriched FC - Principal component 1 - Positive correlation | 17 | 0.081 | 36 | 88 | 52 |
|  |  |  |  |  |  |
|  |  |  |  |  |  |
| 5HT1a enriched FC - Principal component 3 - Negative correlation | 12 | 0.072 | 71 | 54 | 31 |
|  |  |  |  |  |  |
| 5HT1a enriched FC - Complex Imagery - Negative correlation | **251** | **0.005** | **6** | **-62** | **52** |
|  | **190** | **0.038** | **-10** | **-64** | **60** |
|  | 168 | 0.065 | -28 | -52 | 38 |
|  | 40 | 0.05 | -24 | 2 | 58 |
|  | 17 | 0.073 | 38 | -36 | 38 |
|  |  |  |  |  |  |
| 5HT1a enriched FC - Simple Hallucination - Negative correlation | 120 | 0.051 | -18 | -56 | 50 |
|  | 119 | 0.055 | -34 | -44 | 42 |
|  | 15 | 0.089 | 8 | -56 | 54 |
|  |  |  |  |  |  |
| 5HT1a enriched FC - Ego dissolution - Negative correlation | 15 | 0.081 | 64 | -32 | 46 |
|  | 4 | 0.094 | 54 | -34 | 46 |
|  | 1 | 0.099 | -64 | -16 | 22 |
|  |  |  |  |  |  |
| 5HT1b enriched FC - Complex Imagery - Negative correlation | 84 | 0.051 | -30 | -52 | 40 |
|  | 25 | 0.073 | 40 | -48 | 42 |
|  | 8 | 0.09 | 38 | -36 | 36 |
|  |  |  |  |  |  |
| 5HT1b enriched FC - Simple Hallucination - Negative correlation | **126** | **0.024** | **-28** | **-52** | **42** |
